# Effective Glucose Uptake by Human Astrocytes Requires Its Sequestration in the Endoplasmic Reticulum by Glucose-6-Phosphatase-β

**DOI:** 10.1016/j.cub.2018.08.060

**Published:** 2018-11-05

**Authors:** Margit S. Müller, Maxime Fouyssac, Colin W. Taylor

**Affiliations:** 1 Department of Pharmacology, University of Cambridge, Cambridge CB2 1PD, UK; 2 Department of Psychology, University of Cambridge, Cambridge CB2 3EB, UK

**Keywords:** astrocyte, Ca^2+^ signaling, endoplasmic reticulum, genetically encoded glucose sensor, glucose, glucose 6-phosphate, glucose 6-phosphatase

## Abstract

After its uptake into the cytosol, intracellular glucose is phosphorylated to glucose-6-phosphate (G6P), trapping it within the cell and preparing it for metabolism. In glucose-exporting tissues, like liver, G6P is transported into the ER, where it is dephosphorylated by G6Pase-α. The glucose is then returned to the cytosol for export [1, 2]. Defects in these pathways cause glycogen storage diseases [1]. G6Pase-β, an isozyme of G6Pase-α, is widely expressed [3, 4]. Its role in cells that do not export glucose is unclear, although mutations in G6Pase-β cause severe and widespread abnormalities [5, 6, 7]. Astrocytes, the most abundant cells in the brain, provide metabolic support to neurons, facilitated by astrocytic endfeet that contact blood capillaries or neurons [8, 9, 10, 11, 12]. Perivascular endfeet are the main site of glucose uptake by astrocytes [13], but in human brain they may be several millimeters away from the perineuronal processes [14]. We show that cultured human fetal astrocytes express G6Pase-β, but not G6Pase-α. ER-targeted glucose sensors [15, 16] reveal that G6Pase-β allows the ER of human astrocytes to accumulate glucose by importing G6P from the cytosol. Glucose uptake by astrocytes, ATP production, and Ca^2+^ accumulation by the ER are attenuated after knockdown of G6Pase-β using lentivirus-delivered shRNA and substantially rescued by expression of G6Pase-α. We suggest that G6Pase-β activity allows effective uptake of glucose by astrocytes, and we speculate that it allows the ER to function as an intracellular “highway” delivering glucose from perivascular endfeet to the perisynaptic processes.

## Highlights

• Glucose-6-phosphatase-β (G6Pase-β) is expressed in human astrocytes
• G6P is sequestered by ER and dephosphorylated to glucose in the lumen by G6Pase-β
• Loss of G6Pase-β reduces glucose uptake, intracellular ATP, and ER Ca^2+^ content
• ER may provide a protected highway for long-range glucose transport in astrocytes

## Graphical Abstract

**Figure:**
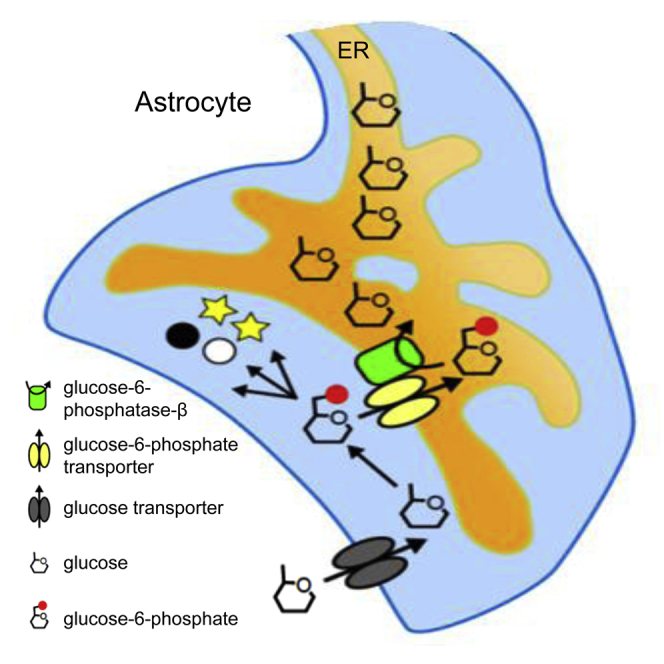

## Teaser

Müller et al. use targeted glucose sensors to show that glucose-6-phosphate uptake by the endoplasmic reticulum of human astrocytes and its dephosphorylation in the ER by glucose-6-phosphatase-β deliver glucose to the ER lumen and sustain cellular glucose uptake. The ER lumen may provide an intracellular protected highway for glucose transport.

## Results

### The ER of Human Astrocytes Sequesters Glucose by Uptake and Dephosphorylation of Gluose-6-Phosphate

**Figure 1:**
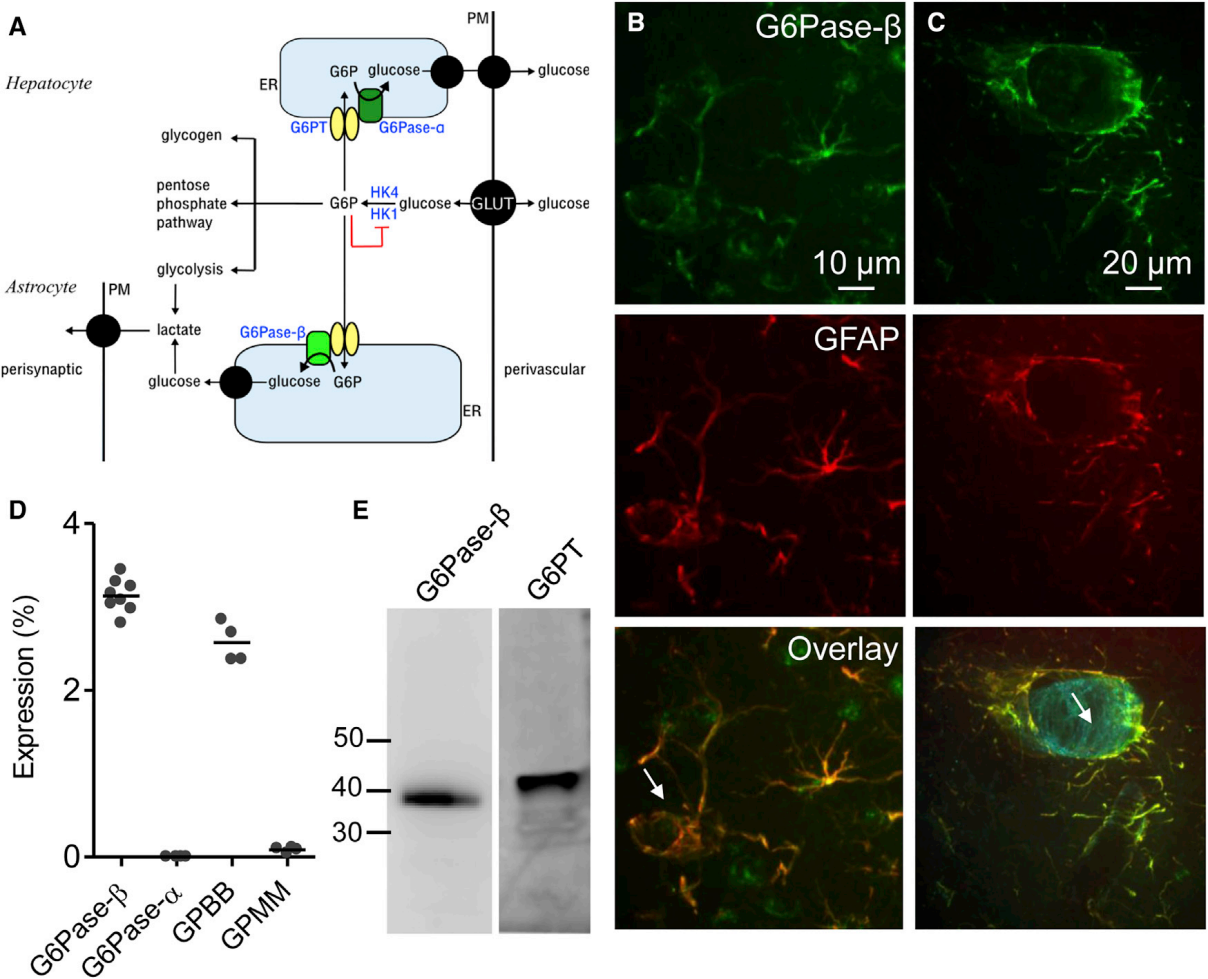
G6Pase-β Is Expressed in Astrocytes from Rodents and Humans (A) Glucose transported into cells by glucose transporters (GLUT) is phosphorylated to G6P by HK. Glucose-exporting cells, like hepatocytes, use a G6P transporter (G6PT) to transport G6P into the ER, where it is dephosphorylated by G6Pase-α and then exported from the cell, possibly at ER-PM contact sites (top right). G6PT and G6Pase-α are viewed as adaptations that allow efficient glucose export. Astrocytes are proposed to provide neurons with a source of energy and neurotransmitter precursors by importing glucose at their perivascular endfeet, glycolytically metabolizing it, and then exporting neurotransmitter precursors and perhaps lactate at perisynaptic processes [17]. Although the importance of the lactate shuttle has been questioned [10, 11, 12], it is clear that astrocytes provide metabolic support to neurons. Our results suggest that G6PT and G6Pase-β allow the ER of astrocytes to serve as an intracellular highway moving glucose from perivascular endfeet to perisynaptic processes. (B and C) Confocal z stacks of rat brain cortical slices immunostained for G6Pase-β and GFAP showing their colocalization. Arrows in the overlays indicate the lumen of blood vessels surrounded by astrocytes. The overlay in (C) is additionally stained with isolectin B4 to identify capillaries (blue). (D) qPCR showing expression levels of mRNA (relative to GAPDH) of the indicated enzymes in human astrocytes: GPBB and GPMM are two isoforms of glycogen phosphorylase. Results show each independent determination (n = 4–8 isolates, derived from at least 4 different cultures) and the mean. (E) Immunoblots (30 μg protein/lane), typical of 3 similar blots from independent treatments, show expression of G6Pase-β and G6PT in human astrocytes.

The contribution of G6Pase-α to glucose handling in tissues like liver that export glucose is clear (Figure 1A). G6Pase-β, which is more widely expressed, has a similar structure to G6Pase-α, and it is expressed in endoplasmic reticulum (ER) membranes with a luminally disposed catalytic site, but its function is unknown [3, 4]. The only brain cells reported to express G6Pase-β are rodent astrocytes [18]. We confirmed, by immunostaining of cortical slices from adult rat brain, that G6Pase-β is selectively expressed in astrocytes (Figures 1B and 1C). Using qPCR and/or immunoblotting, we established that a G6P transporter (G6PT, see Figure 1A) and G6Pase-β, but not G6Pase-α, are also expressed in cultured human astrocytes (Figures 1D and 1E). Subsequent experiments examine the contribution of G6Pase-β to glucose homeostasis and energy metabolism in normal human astrocytes derived from the fetal cortex.

**Figure 2:**
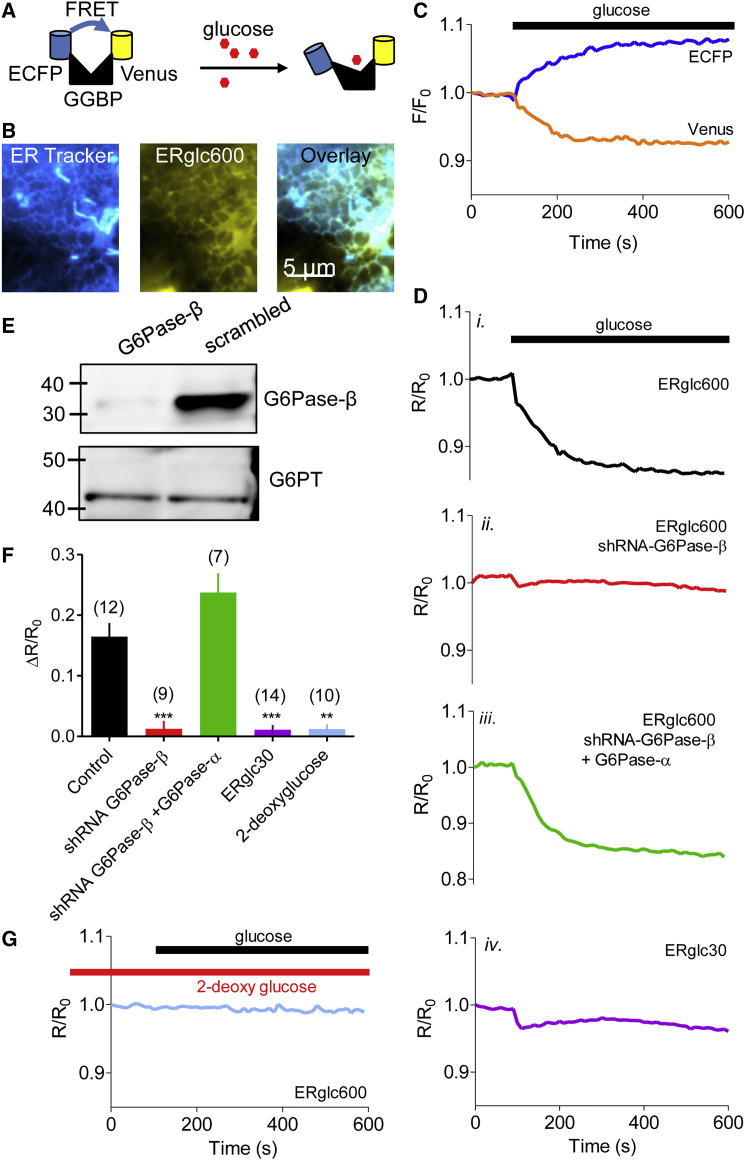
The ER of Human Astrocytes Sequesters Glucose by Importing G6P (A) The ER glucose-sensors comprise a glucose-binding protein (GGBP) tethered to enhanced cyan fluorescent protein (ECFP) and Venus, such that glucose binding separates the chromophores causing a decrease in FRET efficiency. (B) Total internal reflection fluorescence (TIRF) image of astrocyte showing colocalization of ER-Tracker Red (shown in blue) with ERglc600 (yellow). (C) Typical trace from a single astrocyte expressing ERglc600 and exposed to glucose (5 mM, bar) showing reciprocal changes in the fluorescence of Venus and ECFP (F/F_0_, where F_0_ is the fluorescence recorded before adding glucose). (D) FRET ratios (R/R_o_, Venus/ECFP) were recorded using ERglc600 (*i*, *ii*, *iii*) or ERglc30 (*iv*) after adding glucose (5 mM) to normal astrocytes (*i*, *iv*), after shRNA-mediated knockdown of G6Pase-β (*ii*) alone or with expression of G6Pase-α (*iii*). (E) Western blots (WBs) (30 μg protein/lane), typical of 3 independent transfections, show effects of G6Pase-β shRNA and scrambled shRNA on expression of G6Pase-β and G6PT. Positions of M_r_ markers (kDa) are shown. (F) Summary results (mean ± SEM from (*n*) independent cells; *n* shown above bars) show R/R_o_ determined 250 s after addition of glucose or 2-deoxyglucose. ^∗∗∗^p < 0.001, ^∗∗^p < 0.01, Kruskal-Wallis with Dunn’s multiple comparisons test, relative to control. (G) Analysis of astrocytes expressing ERglc600 and pretreated with 2-deoxglucose (5 mM, 30 min) to inhibit HK before addition of glucose (5 mM).

We used ER-targeted glucose sensors with high (${\text{K}}_{\text{D}}^{\text{glucose}}$ = 30 μM, ERglc30) or low affinity for glucose (${\text{K}}_{\text{D}}^{\text{glucose}}$ = 600 μM, ERglc600) [15, 16] to measure glucose concentrations within the ER of cultured human astrocytes (Figures 2A and 2B). The sensors selectively detect glucose, but not G6P or 2-deoxyglucose [19]. Restoration of extracellular glucose (5 mM) to astrocytes incubated in glucose-free medium for 10 min caused a decrease in the fluorescence resonance energy transfer (FRET) ratio of ERglc600, consistent with a sustained increase in ER glucose concentration (Figures 2C and 2Di). There was, however, no change in the FRET ratio of the high-affinity glucose sensor (ERglc30) (Figure 2Div). This suggests that ERglc30 is already saturated with glucose. Since ERglc30 and ERglc600 differ only within the glucose-binding site [15, 16], it further demonstrates that the FRET changes recorded with ERglc600 are due to changes in glucose concentration rather than to other effects, for example, pH changes or changes in the fluorescence of endogenous metabolites.

Lentivirus-mediated delivery of appropriate short hairpin RNA (shRNA) effectively reduced expression of G6Pase-β without affecting G6PT (Figure 2E). Loss of G6Pase-β abolished accumulation of glucose by the ER (Figures 2Dii and 2F, see legends for statistical analyses). We used human G6Pase-α for rescue experiments since it has the same catalytic activity and ER expression as G6Pase-β, but it is normally expressed only in liver and kidney. Expression of G6Pase-α rescued ER glucose uptake in cells lacking G6Pase-β (Figures 2Diii and 2F). Preincubation of astrocytes with 2-deoxyglucose to inhibit hexokinase (HK, see Figure 1A) abolished accumulation of glucose by the ER (Figures 2F and 2G).

**Figure 3:**
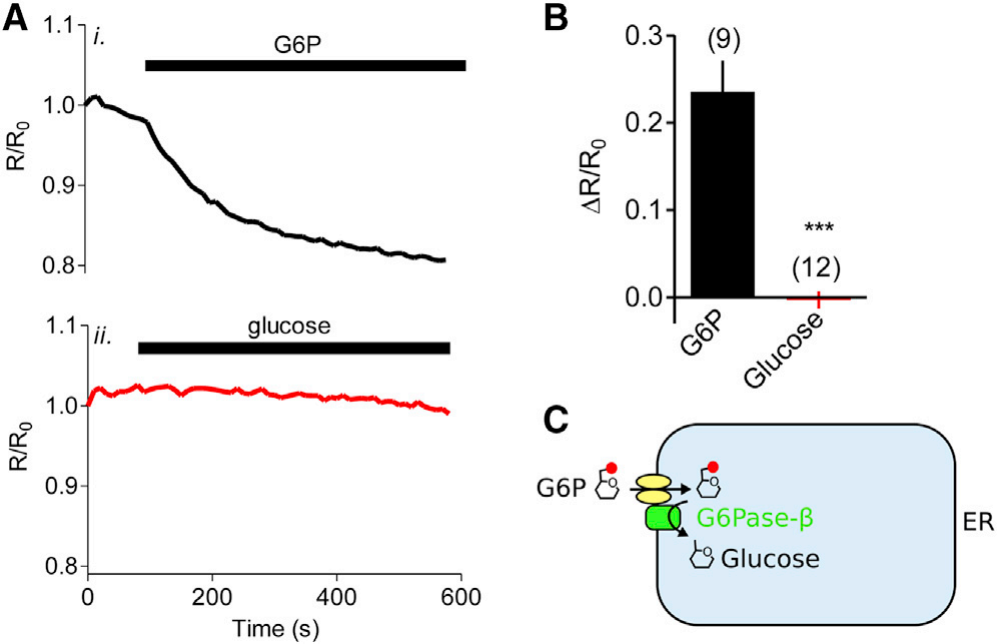
The ER of Permeabilized Astrocytes Accumulates G6P but Not Glucose (A) FRET ratios (R/R_o_) were recorded from individual permeabilized astrocytes expressing ERglc600 after addition of G6P (5 mM, *i*) or glucose (5 mM, *ii*). (B) Summary results (mean ± SEM from [*n*] independent cells, *n* shown above bars) show R/R_o_ determined 250 s after addition of glucose or G6P. ^∗∗∗^p < 0.001, Mann-Whitney test. (C) The results show that the ER of astrocytes accumulates glucose by import, and then dephosphorylation, of G6P, rather than by directly transporting glucose.

The results so far show that both HK and G6Pase-β are required for the ER to sequester glucose, suggesting that the ER may import G6P from the cytosol using G6PT, and then use the luminal catalytic site of G6Pase-β to dephosphorylate G6P to glucose (Figure 1A). We tested this directly using astrocytes in which the plasma membrane was permeabilized by digitonin. Addition of G6P, but not of glucose, to permeabilized astrocytes caused accumulation of glucose within the ER (Figures 3A and 3B). This observation excludes the possibility that, in intact cells, cytosolic glucose reaches the ER lumen passively or through glucose transporters. These results demonstrate that G6Pase-β is required for uptake of glucose, imported as G6P, by the ER of human astrocytes (Figure 3C). This is consistent with an analysis of rodent astrocyte microsomes, where G6P uptake was attenuated in mice lacking G6PT, but unaffected by loss of G6Pase-α [18].

### G6Pase-β Is Required for Glucose Uptake, ATP Production, and Ca^2+^ Uptake by ER

**Figure 4:**
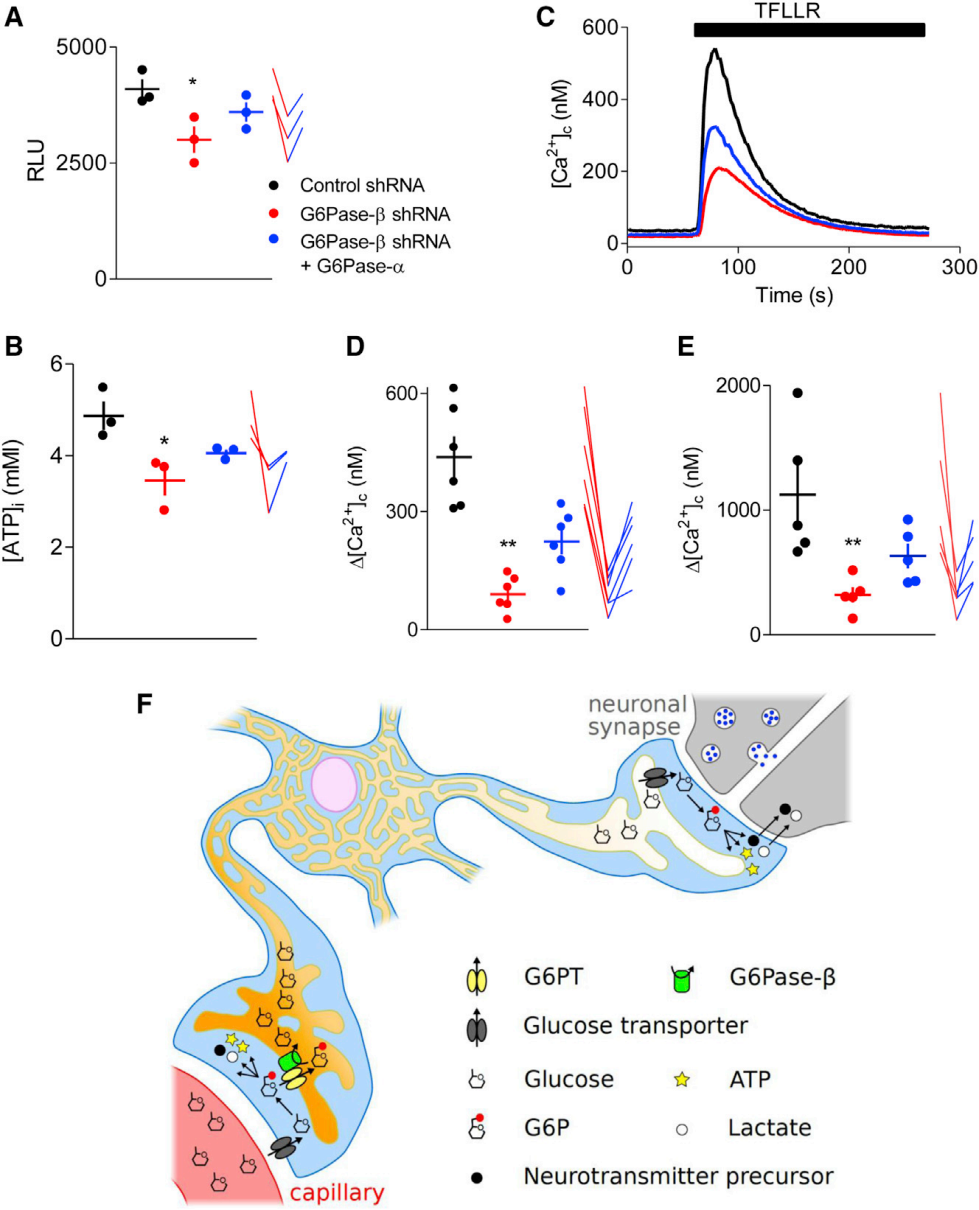
G6Pase-β Is Required For Glucose Uptake, ATP Synthesis, and ER Ca^2+^ Accumulation (A) Effects of the indicated shRNA alone or after expression of G6Pase-α on 2-deoxyglucose uptake (10 mM, 10 min) by populations of astrocytes. Results show individual values and mean ± SEM (n = 3 independent treatments). For clarity, the before-after treatments for each set of determinations are shown alongside by lines. Color code applies to all panels. RLU, relative luminescence units. For all summary data, ^∗∗^p < 0.01, ^∗^p < 0.05, Friedman test with Dunn’s multiple comparisons test, relative to control. (B) Effects of the same treatments on intracellular ATP (individual values, and mean ± SEM, n = 3 independent treatments). (C) Typical traces (duplicate measurements) show Ca^2+^ signals evoked by stimulation of PAR 1 by TFLLR (30 μM) in HBS for astrocytes treated as indicated. (D and E) Summary results shown peak increases in [Ca^2+^]_c_ (Δ[Ca^2+^]_c_) evoked by TFLLR in HBS (D) or ionomycin (1 μM) in Ca^2+^-free HBS (E). The latter to determine the Ca^2+^ content of the intracellular stores. Results show individual values and means ± SEM, n = 6 (D) or 5 (E) independent analyses, each with duplicate determinations. (F) The ER of astrocytes provides an intracellular highway for glucose transport. Glucose from capillaries is transported into the perivascular endfeet of astrocytes by GLUT1 and then phosphorylated by HK to G6P, which is then metabolized in the cytosol or transported into the ER by the G6P transporter (G6PT). Within the ER, the luminal catalytic site of G6Pase-β dephosphorylates G6P to glucose. Hence, G6Pase-β both ensures removal of G6P from the cytosol, where its accumulation would inhibit HK and prevents further glucose uptake, and it delivers glucose to the ER lumen, where it is protected from further metabolism and free to diffuse. An ER glucose transporter can then return glucose to the cytosol, where its phosphorylation to G6P by HK allows it to enter glycolysis. This then provides ATP within the perisynaptic process and lactate and neurotransmitter precursors for export to neurons. Hence, G6Pase-β within the ER allows effective glucose uptake at perivascular endfeet and its transport through a protected ER highway to perisynaptic processes.

Knockdown of G6Pase-β reduced glucose uptake by astrocytes and their intracellular ATP concentration, and both effects were partially rescued by expression of G6Pase-α (Figures 4A and 4B). We examined inositol 1,4,5-trisphosphate (IP_3_)-mediated Ca^2+^ release from the ER to explore the functional consequence of losing G6Pase-β. Astrocytes were stimulated with TFLLR, a peptide agonist of the protease-activated receptor 1 (PAR 1), which is coupled to G_q_ and thereby formation of IP_3_ [20]. Loss of G6Pase-β reduced the amplitude of the PAR 1-evoked increase in cytosolic free Ca^2+^ concentration ([Ca^2+^]_c_) (Figures 4C and 4D). Several steps between PAR1 and the increase in [Ca^2+^]_c_ require ATP, including G-protein activation, formation of IP_3_, regulation of IP_3_ receptors by ATP, and the activities of plasma membrane (PMCA) and ER (SERCA) Ca^2+^-ATPases. We examined SERCA because it has been reported to rely on glycolysis-derived ATP, requiring both glucose uptake and glycogen degradation [21, 22]. Loss of G6Pase-β reduced the Ca^2+^ content of the ER, assessed using ionomycin, by a similar amount (∼70%, Figure 4E) to the decrease in amplitude of the PAR1-evoked Ca^2+^ signals (Figure 4D). The effects of knocking down G6Pase-β were partially rescued by expression of G6Pase-α (Figure 4). We have not examined the effects of G6Pase-β on other steps in the signaling sequence, but its effects on Ca^2+^ uptake by the ER seem sufficient to account for the diminished Ca^2+^ signals evoked by PAR 1.

## Discussion

Glucose occupies a central position in the metabolism of all eukaryotic cells because it is a major energy source and a precursor for many metabolic intermediates. The capacity of most cells to sequester glucose is constrained by the properties of their HK (HK I), which, unlike the isoform expressed in liver (HK IV) [23], is saturated by prevailing glucose concentrations and feedback inhibited by G6P [24]. Glucose meets the metabolic needs of astrocytes, and it allows them to support neurons by providing them with intermediates for metabolism and neurotransmitter synthesis. A popular hypothesis suggests that lactate is exported from astrocytes to meet these needs [17], but the importance of this “lactate shuttle” is disputed [10, 11, 12]. It is, however, clear that neuronal activity stimulates glucose uptake at the perivascular endfeet of astrocytes [25, 26, 27], where the glucose transporter (GLUT1) is enriched [13]. Effective support of neurotransmission by astrocytes therefore requires both efficient glucose uptake at the perivascular endfeet, and its transfer across considerable distances, several millimeters in human brain [14], to perisynaptic processes.

We have shown that the ER of human astrocytes accumulates glucose by sequestering G6P. This requires G6Pase-β, and disrupting the sequence inhibits cellular glucose uptake, ATP production, and ER Ca^2+^ uptake. In neutrophils and macrophages too, loss of G6Pase-β reduced glucose uptake, lactate, and ATP production and attenuated Ca^2+^ signaling [28, 29]. We suggest two important roles for G6Pase-β in astrocytes. First, G6Pase-β sustains cellular glucose uptake by rapidly sequestering the cytosolic G6P that would otherwise inhibit HK and thereby reduce the gradient for glucose influx (Figure 1A) [30, 31, 32]. Evidence that diffusion of a fluorescent analog of 2-deoxyglucose is very restricted within perivascular endfeet [33] lends credence to our suggestion that G6P is likely to accumulate at sites of glucose uptake unless it is sequestered by the ER. The only other means of removing cytosolic G6P, namely, glycolysis, the pentose phosphate pathway, and glycogen synthesis are poorly suited to the task because their regulation is not tuned to the G6P concentration. Second, we suggest that G6Pase-β provides astrocytes with both a second reservoir of glucose (additional to their glycogen stores) [34] and a route by which glucose can pass through the cell protected from glycolytic enzymes (Figure 4F). The ER lumen provides a route, a “Ca^2+^ tunnel,” for redistributing Ca^2+^ within cells [35]. We propose that the ER fulfills a similar function for intracellular glucose transport, with G6PT transporting G6P into the ER lumen, where it is de-phosphorylated by G6Pase-β to generate free glucose. Since glucose is not further metabolized within the ER, it can then diffuse unhindered through the ER lumen until it encounters a glucose transporter. In permeabilized cells, glucose does not readily enter the ER (Figure 3A), suggesting that such transporters may either selectively transport glucose out of the ER or their overall activity is low, consistent perhaps with them being concentrated at glucose-export sites (Figure 1A). After translocation back into the cytosol, glucose may be phosphorylated to G6P and then further metabolized to support the local needs of astrocytes for ATP, and to provide neurotransmitter precursors and lactate for export to neurons (Figures 1A and 4F) [36]. This intracellular glucose highway may function in many cells, but it is likely to be particularly important for astrocytes delivering glucose acquired at their perivascular endfeet to their perisynaptic processes that may be several millimeters away (Figure 4F).

We conclude that G6Pase-β, a widely expressed but hitherto enigmatic enzyme, fulfills two essential roles in astrocytes. By allowing rapid sequestration of G6P at perivascular endfeet, it allows sustained glucose uptake, and by delivering glucose to the ER lumen it provides a protected intracellular highway for effective delivery of glucose from perivascular endfeet to perisynaptic processes (Figures 1A and 4F). The complex architecture of astrocytes, where the major sites of glucose uptake and demand are widely separated, may exaggerate their need for G6Pase-β, but it seems likely that, in other cells too, G6Pase-β may both facilitate glucose uptake and its intracellular distribution.

## STAR★Methods

### Key Resources Table

**Table:** 

REAGENT OR RESOURCE	SOURCE	IDENTIFIER
**Antibodies**		

IC, immunocytochemistry; WB, western blot		
Rabbit anti-G6Pase-β (IC)	Santa Cruz Biotechnology, Dallas, USA	Cat#sc-134714; RRID:AB_10647643
Rabbit anti-G6PT (WB)	Santa Cruz Biotechnology	Cat#sc-135479; RRID:AB_2254868
Donkey anti-mouse IgG-HRP (WB)	Santa Cruz Biotechnology	Cat#sc-2314; RRID:AB_641170
Donkey anti-rabbit IgG-HRP (WB)	Santa Cruz Biotechnology	Cat#sc-2077; RRID:AB_631745
Goat anti-GFAP (IC)	AbCam, Cambridge, UK	Cat#ab 53554; RRID:AB_880202
Rabbit anti-G6Pase-α (WB)	AbCam	Cat#ab 83690; RRID:AB_1860503
Rabbit anti-G6Pase-β (WB)	AbCam	Cat#ab 133964
Donkey anti-rabbit AlexaFluor 488 (IC)	ThermoFisher, Paisley, UK	Cat#A-21206; RRID:AB_141708
Donkey anti-goat AlexaFluor 633 (IC)	ThermoFisher	Cat#A-21082; RRID:AB_141493

**Bacteria and Virus Strains**		

Lentiviral transfer vector, FUGW	Addgene	Addgene#14883
Envelope vector, pMD2.G	Addgene	Addgene#12259
Packaging vector, pMDLg/pRRE	Addgene	Addgene#12251
Packaging vector, pRSV.Rev,	Addgene	Addgene#12253
Packaging vector, psPAX2	Addgene	Addgene#12260

**Chemicals, Peptides, and Recombinant Proteins**		

ATP	Sigma-Aldrich, Gillingham, UK	Cat#A9187
2-deoxy-d-glucose	Sigma-Aldrich	Cat#D6134
AlexaFluor 568-conjugated isolectin GS-B_4_	ThermoFisher	Cat#121412
Astrocyte growth medium (AGM BulletKit)	Lonza, Slough, UK	Cat#CC 3186
BAPTA	Molekula, Dorset, UK	Cat#20358510
Bovine serum albumin (BSA)	Europa Bioproducts Ltd, Cambridge, UK	Cat#EQBAH64
Cal-520AM	AAT Stratech Scientific, Suffolk, UK	Cat#21130
Dimethyl sulfoxide (DMSO)	Sigma-Aldrich	Cat#D2650
ECL Prime reagents	GE Healthcare Life Sciences, Little Chalfont, UK	Cat#RPN2232
ER Tracker Red	ThermoFisher	Cat#E34250
Fibronectin	Merck Millipore, Watford, UK	Cat#FC010
Fetal bovine serum (FBS)	Sigma-Aldrich	Cat#F7524, batch 094M3341
d-glucose-6-phosphate (G6P)	Sigma-Aldrich	Cat#G7879
Ionomycin	Apollo Scientific, Bredbury, UK	Cat#56092-81-0
Lipofectamine LTX	ThermoFisher	Cat#15338100
PAR-1 peptide, TFLLR	Tocris, Bristol, UK	Cat#1464
Probenecid	Sigma-Aldrich	Cat#P8761
Triton X-100	Sigma-Aldrich	Cat#T8787

**Critical Commercial Assays**		

ATP assay kit	AbCam	Cat#ab113849
Glucose Uptake-Glo assay kit	Promega Southampton, UK	Cat#J1341
Fastlane cell cDNA kit	QIAGEN	Cat#215011

**Experimental Models: Cell Lines**		

293FT cells	ThermoFisher	Cat#R70007
Human astrocytes from fetal cortex	Lonza, Slough, UK	Cat#CC-2565

**Recombinant DNA**		

Plasmid (pEF/myc/ER FLIPglu-600uDelta13V) encoding ERglc600	Addgene	Addgene#18020
Plasmid (pEF/myc/ER FLIPglu-30uDelta13V) encoding ERglc30	Addgene	Addgene#18021
CCSB-Broad LentiORF-G6Pase-α	Dharmacon, Lafayette, USA	Cat#ccsbBroad304_04582
TRC human G6Pase-β shRNA	Dharmacon	Cat#TRCN0000051500
Quantitect primer assay: GAPDH	QIAGEN, Crawley, West Sussex, UK	Cat#Hs_GAPDH_1_SG
Quantitect primer assay: G6Pase-β	QIAGEN	Cat#Hs_G6PC3_1_SG
Quantitect primer assay: G6Pase-α	QIAGEN	Cat#Hs_G6PC_1_SG
Quantitect primer assay: GPBB	QIAGEN	Cat#Hs_PYGB_1_SG
Quantitect primer assay: GPMM	QIAGEN	Cat#Hs_PYGM_1_SG
Quantitect primer assay: GFAP	QIAGEN	Cat#Hs_GFAP_1_SG

**Software and Algorithms**		

Prism 5	GraphPad, La Jolla, USA	https://www.graphpad.com/

### Contact for Reagent and Resource Sharing

Further information and requests for resources and reagents should be directed to and will be fulfilled by Colin W. Taylor (cwt1000@cam.ac.uk)

### Experimental Model and Subject Details

Normal human astrocytes from fetal cortex were supplied as frozen cells that had not been passaged (catalog number CC-2565, Lonza, Slough, UK). The cells were confirmed, by Lonza, to be free of infection with HIV-1 and hepatitis B and C, and we confirmed they were free of mycoplasma. Astrocytes were grown at 37°C in humidified air containing 5% CO_2_, using astrocyte growth medium (AGM BulletKit) supplemented with 3% fetal bovine serum. AGM includes human epidermal growth factor, insulin, ascorbic acid, gentamycin and l-glutamine. Cells were passaged using trypsin when they reached 70%–80% confluence.

Outbred male Sprague Dawley rats (aged 9-10 weeks, 320-350 g, Charles River Laboratories, Kent, UK) were immunocompetent, and after screening by Charles River Laboratories, their health profile was categorized as VAP/Plus^®^. Rats were habituated to the colony for one week in a reversed light-dark cycle (lights off at 7 a.m) with *ad libitum* access to food and water. They were not subject to drug treatments or procedures prior to being euthanised with pentobarbital (300 mg, Dolethal, Vetoquinol UK Ltd, Buckingham, UK) and perfused transcardially with isotonic saline followed by 10% neutral buffered formalin (Sigma). Brains were transferred to 20% sucrose solution with 1% PBS, and 30-μm coronal sections were prepared after 24 h. Experiments complied with the United Kingdom 1986 Animals (Scientific Procedures) Act, after ethical review by the University of Cambridge Animal Welfare and Ethical Review Body.

### Method Details

#### Lentiviral Vectors

Lentiviral vectors were used to express ER-targeted glucose sensors and a short-hairpin RNA (shRNA) against G6Pase-β. Plasmids encoding the glucose sensors, ERglc600 and ERglc30 [15, 16] were subcloned into the lentiviral transfer vector FUGW by PCR-based cloning. Lentiviral particles for sensors and shRNA were produced in 293FT cells by transfection with equal amounts of transfer vector (TRC human G6Pase-β shRNA, or FUGW containing ERglc30 or ERglc600), envelope vector (pMD2.G), and two packaging vectors (pMDLg/pRRE and pRSV.Rev), using Lipofectamine LTX. Lentiviral particles of CCSB-Broad LentiORF-G6Pase-α were produced using one packaging vector (psPAX2). Viral supernatant was collected after 48 h, filtered (0.45 μm), incubated with LentiX concentrator (16 h at 4°C, Clontech), collected (1500 x*g*, 45 min, 4°C) and the pellets were re-suspended in AGM at 10% of the original volume, and stored at −80°C. For expression of fluorescent sensors or G6Pase-α, and knockdown of G6Pase-β, astrocytes were incubated with lentiviral particles (multiplicity of infection, MOI = 2) in complete AGM, and used after 72 h.

#### Measurements of [Ca^2+^]_c_

For measurements of [Ca^2+^]_c_, confluent cultures of astrocytes grown in fibronectin-coated 96-well plates (Greiner Bio-One, Stonehouse, UK) were incubated at 20°C with Cal-520AM (2 μM) in HBS containing probenecid (2.5 mM) [37]. HBS comprised: 135 mM NaCl, 5.9 mM KCl, 1.2 mM MgCl_2_, 1.5 mM CaCl_2_, 11.5 mM glucose and 11.6 mM HEPES, pH 7.3. After 60 min, cells were washed, incubated in HBS (90 min), washed and used for experiments at 20°C in HBS. Ca^2+^-free HBS contained BAPTA (2.5 mM), which was added immediately before stimulation to reduce the free [Ca^2+^] of HBS to < 100 nM. Fluorescence (excitation at 490 nm, emission at 520 nm) was recorded at 1.44 s intervals using a FlexStation III fluorescence plate-reader (MDS Analytical Technologies, Wokingham, UK) [38]. Fluorescence (F) was calibrated to [Ca^2+^]_c_ from: ${\left[{\text{Ca}}^{2+}\right]}_{\text{c}}={\text{K}}_{\text{D}}\frac{\text{F}-{\text{F}}_{\text{min}}}{{\text{F}}_{\text{max}}-\text{ F}}$, where K_D_ is the equilibrium dissociation constant of Cal-520 for Ca^2+^ (320 nM), F_min_ and F_max_ are the minimal and maximal fluorescence values determined after addition of Triton X-100 (0.2% v/v) in Ca^2+^-free HBS (F_min_) or ionomycin (10 μM) in HBS (F_max_). In these, and all other analyses of cells grown in multi-well plates, the distribution of treatments across wells was systematically changed between replicate experiments to avoid potential position-related artifacts.

#### Western Blotting

Confluent cultures of astrocytes grown in 6-well plates were scraped into lysis medium (150 mM NaCl, 0.5 mM EDTA, 1% Triton X-100, 10 mM Tris/HCl pH 7.5, Pierce protease inhibitor mini-tablet with EDTA, 1 tablet/10 ml, 4°C). After 1 h, lysates were sonicated (Transonic ultrasonic bath, 3 × 10 s) and the supernatant was recovered (20,000 x*g*, 30 min). Proteins were separated on NuPAGE 3%–8% Tris-acetate gels and transferred to iBlot PVDF membranes using an iBlot gel-transfer device (ThermoFisher). Membranes were blocked by incubation (1 h) with Tris-buffered saline (TBS: 137 mM NaCl, 20 mM Tris, pH 7.6) containing BSA (5%) and Tween-20 (0.1%), incubated (12 h, 4°C) with primary antibody in blocking buffer, washed in TBS (3 × 15 min), and incubated (1 h, 20°C) with HRP-conjugated secondary antibody in blocking buffer. After washing in TBS (3 × 15 min) antibodies were detected using ECL Prime reagents and a PXi luminescence imaging system. Antibodies were diluted as follows: G6PT (1:500), G6Pase-α (1:100), G6Pase-β (1:500), and HRP-conjugated secondary antibodies (1:5000).

#### Immunocytochemistry

Rat cortical brain slices were washed in PBS, incubated (20°C, 15 min) in blocking buffer (PBS with 10% BSA and 0.3% Triton X-100), washed in PBS (3 × 5 min), and incubated (72 h, 4°C) with primary antibodies or isolectin B4 in PBS containing 0.1 mM CaCl_2_, 0.05% Triton X-100 and 1% BSA. After washing in PBS (3 × 30 min), slices were incubated with secondary antibodies (16 h, 4°C), washed in PBS (3 × 30 min), and imaged. Antibodies and lectin were diluted as follows: G6Paseβ (1:50), GFAP (1:1000), isolectin B4 (1:50) and secondary antibodies (1:10000).

#### Measurements of Glucose Uptake and Intracellular ATP

Confluent cultures of astrocytes in 96-well plates at 20°C were used to measure intracellular ATP using a luciferin-based ATP assay kit. A Glucose Uptake-Glo assay kit was used to report glucose uptake (by measurement of 2-deoxyglucose uptake) in cells washed with glucose-free HBS before incubation (10 min, 20°C) with 2-deoxyglucose (1 mM). We confirmed that none of the treatments used affected the number of cells/well.

#### Fluorescence Microscopy

Imaging used an inverted Olympus IX83 microscope with 60x**/**1.3NA and 100x/1.49NA objectives, and a multi-line-laser bank with iLas^2^ targeted illumination system (Cairn, Faversham, Kent, UK). Excitation light was passed through a dichroic mirror for 422 nm (ZT442rdc-UF2, Chroma) and a quad dichroic beam-splitter for other wavelengths (TRF89902-QUAD, Chroma). Emitted light passed through filters (Cairn Optospin) with peaks/bandwidths of 480/40 nm, 525/50 nm, 630/75 nm and 700/75 nm, before capture with an iXon Ultra 897 EMCCD camera (512 × 512 pixels, Andor). We confirmed, using astrocytes expressing single fluorophores, that there was no bleed-through between channels. Confocal stacks were generated using spinning-disc confocal microscopy (70-μm pinhole; X-Light, Crest Optics). MetaMorph Microscopy Automation and Image Analysis Software (Molecular Devices) and Fiji [39] were used for image analysis. Images of cells were background-corrected by subtraction of fluorescence from a region devoid of cells.

#### Measurement of G6P uptake by permeabilized astrocytes

To measure G6P and glucose uptake by permeabilized astrocytes expressing ERglc600, cells were permeabilized by digitonin (30 μM, 2 min, 37°C) in cytosol-like medium (CLM) comprising 140 mM KCl, 2 mM NaCl, 1 mM EGTA, 2 mM MgCl_2_, 20 mM PIPES, pH 7. The permeabilized cells were washed with CLM supplemented with MgATP (1.5 mM) and CaCl_2_ (375 μM, free [Ca^2+^] ∼250 nM), and assays were then conducted in the same medium at 20°C.

#### Quantitative PCR

Astrocytes grown to confluence in 24-well plates were lysed (200 μl cell processing buffer/well), and 4 μl lysate was used to generate cDNA (Fastlane cell cDNA kit). Each qPCR mix contained diluted cDNA (1:5, 5 μl), Rotor-Gene SYBR Green PCR master mix (10 μl), Quantitect primer assay (2 μl) and RNase-free water (3 μl). qPCR was performed on a Rotor-Gene 6000 thermocycler (QIAGEN): a denaturation step (95°C, 5 min) was followed by 40 amplification cycles (5 s at 95°C, 10 s at 60°C), with a melting curve recorded at the end of each run (70°C to 95°C). Expression of mRNA relative to glyceraldehyde 3-phosphate dehydrogenase (GAPDH) was calculated from:

Relative expression = $\frac{{\text{E}}^{-{\text{C}}_{\text{T}}^{\text{PROTEIN}}}}{{\text{E}}^{-{\text{C}}_{\text{T}}^{\text{GAPDH}}}}$, where E is the amplification efficiency, calculated as 10^m^, where m is the average increase in fluorescence for four cycles after the cycle threshold C_T_ for the indicated PCR product [40]. Results are reported as means from cDNA samples independently obtained from at least 4 different cultures. Negative controls (exclusion of reverse-transcriptase in cDNA synthesis, or of primers during the qPCR run) were included in each assay. Quantitect primer assays were used for GAPDH, G6Pase-β, G6Pase-α, GPBB, GPMM and GFAP.

### Quantification and Statistical Analysis

Analyses were performed without blinding or power calculations to predetermine sample sizes. Statistical comparisons used non-parametric tests: the Mann-Whitney test or, for multiple comparisons, the Kruskal-Wallis or Friedman test, each with Dunn’s multiple comparisons test (GraphPad Prism 5, La Jolla, CA). Results are presented as means ± SEM of values from at least 3 independent experiments. Sample sizes (*n*) refer to independent experiments (see legends for details).
